# Endodontic Management of Failed Root Canal Treatment in Teeth With Previously Missed Canals: A Report of Two Cases With Rare Root Canal Morphology

**DOI:** 10.7759/cureus.73466

**Published:** 2024-11-11

**Authors:** Eyad A AlTamimi, Muhammad Atif S Agwan, Muhammad Z Ahmad

**Affiliations:** 1 Department of Conservative Dental Sciences, College of Dentistry, Qassim University, Buraydah, SAU

**Keywords:** dental anomalies, mandibular premolar, maxillary canine, root canal anatomy, root canal configuration, root canal morphology, root canal therapy, two root canals

## Abstract

Teeth may exhibit a wide range of anatomical variations in their roots and root canal systems, making them challenging to treat in endodontic procedures. Generally, maxillary canines and mandibular premolars present with a single root with one canal, and variations are comparatively rare. Achieving a fluid-impervious seal in endodontic therapy requires thorough debridement and three-dimensional obturation of the root canal system, which is considered a complex micro-neurologic procedure. Clinicians may fail to achieve the successful outcome without a comprehensive understanding of the pulp chamber and root canal system anatomy. We present a report of successful management of previously failed root canal treatment of two such cases, a maxillary canine and a mandibular premolar, with a previously missed second canal due to their uncommon root canal morphological presentation. This report also comprehensively reviews the literature, focusing on the clinically significant anatomical variations and detailing techniques for the effective management of such anomalies in endodontic treatment. The review provides an in-depth explanation of each stage of treatment, including preoperative diagnosis, intraoperative identification of morphological variations, and evidence-based modern endodontic treatment approaches. Emphasis is placed on ensuring a thorough understanding of these steps to achieve successful outcomes in the management of complex cases involving root canal anatomical variations.

## Introduction

Endodontics is a specialized field of dentistry focused on the structure, function, and diseases of the dental pulp and periradicular tissues. It integrates both basic and clinical sciences, encompassing the biology of healthy pulp tissue and addressing the causes, diagnosis, prevention, and treatment of pulp-related diseases and conditions affecting the surrounding periradicular areas. Morphological changes in the root canal system of the tooth, as Nair et al. [1] described, may adversely affect the operator’s ability to perform endodontic procedures. It is important for clinicians to be aware of the anatomical variations in the teeth they are treating. Treatment failure may occur if the operator does not adequately shape and clean the root canal system [1]. A careful interpretation of preoperative radiographic records and cone beam computed tomography (CBCT) scans are helpful in understanding the root canal morphology and reasons for persistent endodontic infections [2-3].

The primary factors in successful endodontic treatment outcomes are establishing a treatment plan based on accurate diagnosis and the identification of variations in the number of roots and root canals. The anatomy of the root canal system plays a critical role in determining the appropriate approach to root canal therapy, as it can directly influence the treatment outcome and prognosis [4]. Vertucci divided permanent tooth root canal configurations into eight distinct categories based on the potential branching of the root canal system. The range of the classification was one to three distinct root canals [5]. Maxillary canines and mandibular premolars and canines are more likely to have a single root and a single canal. Cases of two root canals in a permanent mandibular canine are rare, and, in most instances, the two canals merge in the apical third of the root, exiting through a single foramen (Vertucci type-II canal configuration) [3,5].

Slowey suggested that achieving successful endodontic therapy in mandibular premolars can be particularly challenging [6]. A study conducted at the University of Washington evaluated the failure rates of non-surgical root canal treatments across different teeth and found that the mandibular first premolar had the highest failure rate at 11.45% [7]. Additionally, researchers have reported a wide range of incidence rates for mandibular first premolars with two or more root canals, varying from 2.7% to 62.5% [5].

Researchers have documented racial variations in the root canal morphology of maxillary canines in Caucasian, Turkish, and Indian populations [8]. These researchers reported that, even in subjects of the same ethnicity, the anatomical findings varied from region to region. A nationwide study reported anatomic variations among Saudi subjects and concluded that clinicians may benefit from preoperative CBCT scan with a limited field of view to evaluate the patient’s tooth morphology in cases in which periapical radiographs are difficult to interpret [9]. Furthermore, researchers identified Tome’s root as the mandibular first premolar accessory root and observed morphological variations between ethnic groups. They discovered more than 25% of people in Australia and Sub-Saharan Africa had accessory roots. According to Sert and Bayirli, there are differences in the appearance of root canals between the sexes, with women having a higher frequency (44%) of auxiliary roots and canals than men (34%) [10]. This report presents the non-surgical endodontic management of two cases involving previously failed root canal treatments due to missed additional root canals with rare morphological variations in an adult Saudi male patient.

## Case presentation

Case 1

**Figure 1:**
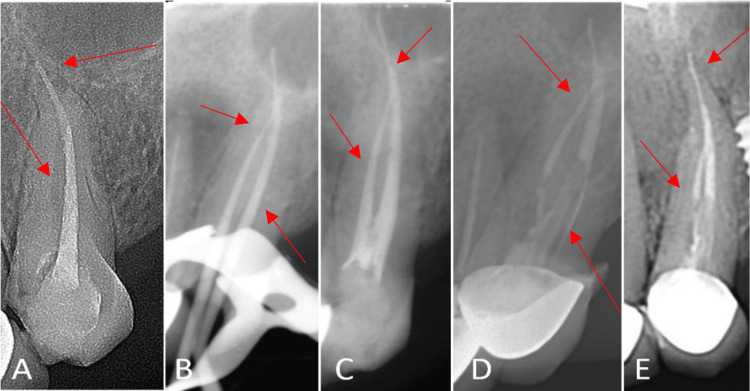
Maxillary canine #23. A. Pre-operative image. B. Master gutta-percha. C. Final obturation. D. Three-month follow-up. E. Six-month follow-up.

A 52-year-old Saudi male patient presented at the dental clinics of the College of Dentistry, Qassim University, Buraydah, Saudi Arabia, with the chief complaint of pain and tenderness in tooth #23. The tooth was periodontally healthy with a normal probing depth of 3 millimeters. Radiographic examination revealed a single-rooted canine. A silhouette was detected along the previously filled canal in #23, revealing a periapical radiolucency (<1 cm) (Figure 1A). A diagnosis of previously failed root canal treatment with symptomatic apical periodontitis was made, with a missed canal identified as the most likely cause of the failure.

After obtaining consent, we administered buccal infiltration of local anesthesia containing 4% articaine hydrochloride with 1:100,000 epinephrine (Ultracaine D-S Forte, Aventis, Istanbul, Turkey) and isolated the tooth using a 6 x 6 rubber dam (Hygenic, Coltene Whaledent, Altstätten, Switzerland) to gain access. We used a sterile round diamond bur (Diatech, Coltene Whaledent) and Endo-Z bur (Dentsply Sirona, Charlotte, NC, USA) with water as coolant in a high-speed rotor (Mani Inc., Tochigi, Japan) and gained access to the pulp chamber space. Previously filled gutta-percha was removed. A K-file #10 was used to find and access the missed root canal, and after radiological verification, the working lengths of the buccal and lingual canals were established to be 25 mm and 18 mm, respectively.

During treatment, an additional canal was discovered, which was found to be connected to the main canal. The previous root filling was removed, and the canals were prepared using the ProTaper Gold (PTG; Dentsply Tulsa Dental Specialties, Johnson City, TN) instrumentation system. We used 5.25% sodium hypochlorite (NaOCl) as an irrigant and also used a 30-G side-perforated closed-ended needle (NaviTip, Ultradent, South Jordan, UT) to deliver 20 mL of irrigant at a rate of 5mL/min in each canal.

We used an apex locator (Tri Auto ZX, J Morita, USA) for working length determination and confirmed it radiographically. We found that both canals join together at the apical third of the root (Type II Vertucci's configuration). We carried out root canal preparation using the crown down technique. The apical part was prepared using hand files (K-files) up to #30 after the coronal preflaring was completed with #4, #3, and #2 gates glidden drills (Dentsply Tulsa Dental). ProTaper instruments were used to clean and shape the canals. A final irrigation with 2% chlorohexidine was performed. We dried the canals using sterile paper points.

We performed the obturation using ProTaper gutta-percha points of size F1 and AH Plus sealer (Dentsply DeTrey GmbH, Konstanz, Germany), employing the cold lateral condensation technique (Figure 1B). The patient was recalled after one week for follow-up. The tooth was asymptomatic (Figure 1C). After post and core placement, a porcelain fused to metal (PFM) crown was placed as the final coronal restoration. At three-month and six-month follow-ups, the tooth showed clinical and radiographic signs of healing (Figures 1D, 1E).

Case 2

**Figure 2:**
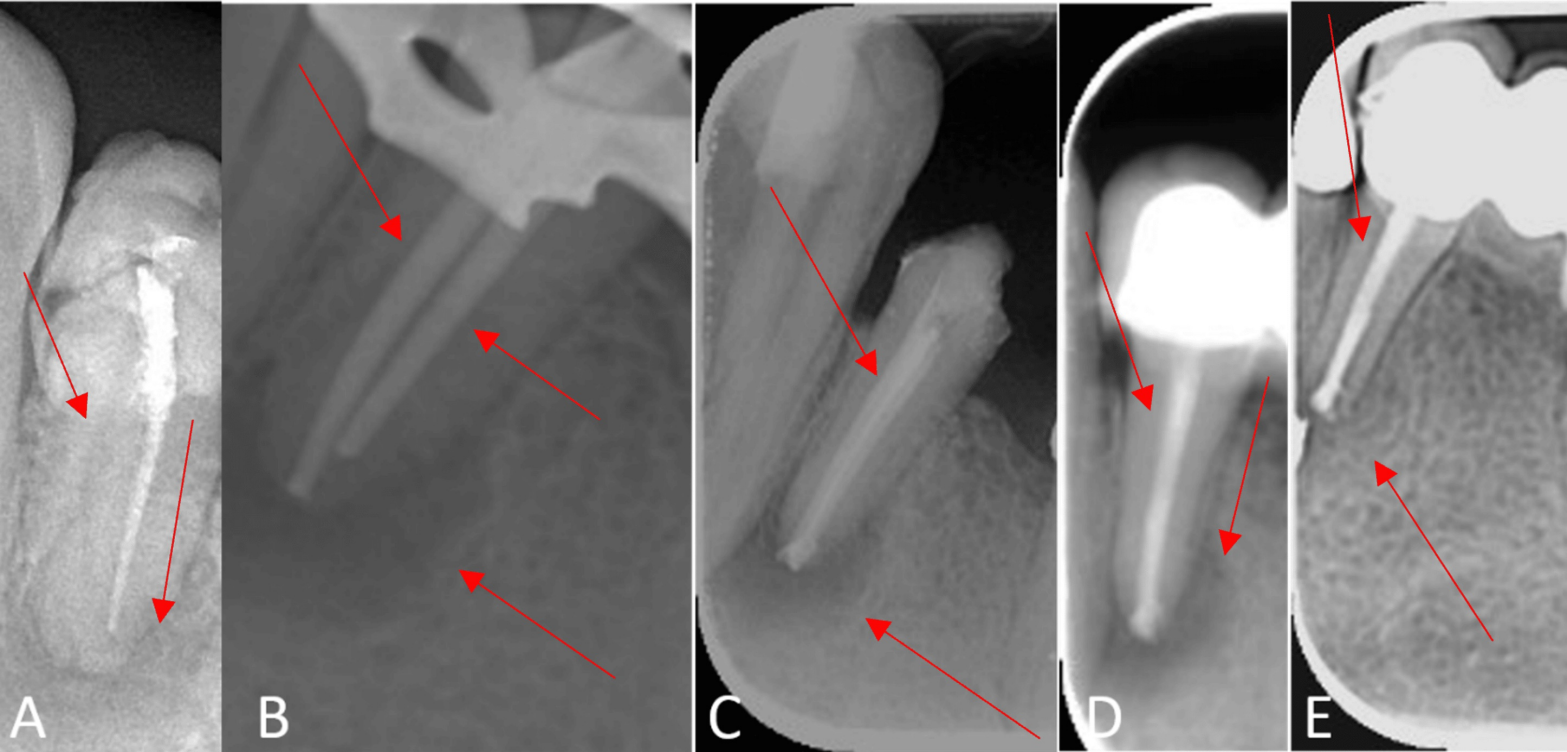
Mandibular first premolar #34. A. Pre-operative image. B. Master gutta-percha. C. Final obturation. D. Three-month follow-up. E. Six-month follow-up.

The same patient as in case 1 presented with pain in tooth #34. A radiographic examination revealed previous root canal treatment with a missed canal and a large periradicular lesion (Figure 2A). A diagnosis of previously failed root canal treatment with symptomatic apical periodontitis was established, with a missed canal identified as the most likely cause of the treatment failure.

We obtained consent similar to case 1 and administered inferior alveolar nerve block using 2 carpules of local anesthesia containing 4% articaine hydrochloride with 1:100,000 epinephrine (Ultracaine D-S Forte, Aventis) and isolated the tooth using a 6 x 6 rubber dam (Hygenic, Coltene Whaledent) to gain access. We used a sterile round diamond bur (Diatech, Coltene Whaledent) and Endo-Z bur (Dentsply Sirona) with water as coolant in a high-speed rotor (Mani Inc., Japan) and gained access to the pulp chamber space. After removing the gutta-percha root canal filling, a previously missed root canal in the same root was found at a lingual position of the previously treated canal. The root canal configuration consisted of two canals originating from the pulp chamber floor and exiting through separate apical foramina (type IV Vertucci's configuration).

We used an apex locator (Tri Auto ZX, J Morita) for working length determination and confirmed it radiographically. The working length was established to be 14 mm for each canal. We copiously irrigated the canals using 5.25% NaOCl as an irrigant. We used a 30-G side-perforated closed-ended needle (NaviTip, Ultradent).

We placed the calcium hydroxide paste as intracanal medicament and sealed the access cavity using sterile dry cotton pallet and light-cured glass ionomer (GC Fuji II LC, GC Corporation, Tokyo, Japan) as temporary restoration (Figure 2B). At the one-week follow-up, the patient was asymptomatic. We removed the intracanal medicament and copiously irrigated the canals with 5.25% NaOCl. A final irrigation was performed with 2% chlorohexidine, and canals were dried using sterile paper points. We obturated the canals using the cold lateral condensation technique (Figure 2C). Post and core were placed. A PFM crown was placed as a final coronal restoration. At three- and six-month follow-up visits, the tooth had radiographic and clinical signs of healing (Figures 2D, 2E).

## Discussion

The accurate diagnosis and identification of the total number of roots and root canals in a tooth prior to treatment are essential for the success of endodontic procedures. Research has indicated that the inability to locate and adequately fill a canal is a significant contributing factor to the failure of nonsurgical endodontic therapy [11].

The presence of two roots in a maxillary canine is uncommon, while the occurrence of more than one canal is exceedingly rare [12-13]. There have been only a few documented cases of a maxillary canine exhibiting two separate root canals [3]. Maxillary canines in the Saudi population showed one root (100%). Of these, 99.0% had one canal, while 1.0% had two canals. Similarly, 99.0% of the teeth had Vertucci’s type I and 1.0% had Vertucci’s type III [14]. On the contrary, the morphology of mandibular premolars exhibited the greatest variability among all dental structures. In the Saudi population, mandibular premolars predominantly presented with a single root (84.8% and 96.1%, respectively) and typically contained one root canal (70.8% and 90.2%, respectively). Conversely, the occurrence of two root canals was relatively rare, observed in only 2.3% and 1.8% of the population, respectively [15].

Intraoral periapical radiographs are essential for understanding the internal anatomy of teeth. Taking multiple angled radiographs can help identify unusual anatomical variations, such as extra roots or canals. However, the limitations of periapical radiography, such as superimposition and the production of two-dimensional images, mean that these methods should be used cautiously in cases involving unusual anatomy [16].

In recent years, CBCT has emerged as a valuable advancement in the field of endodontics. This technology facilitates the identification of anatomical variations in teeth, including deviations from normative structures, curvature, and bifurcation. Furthermore, CBCT provides accurate measurements of tooth length in both sagittal and axial planes. Compared to conventional radiographs, CBCT enhances diagnostic capabilities and contributes to improved outcomes in traditional root canal therapy [17].

## Conclusions

This case report emphasizes the significance of awareness regarding anatomical variations in teeth morphology and serves as a reminder of the exceptional care required to identify and treat additional canals. Clinicians must be vigilant about the anatomical variations present in the teeth they manage and avoid presumptions that canal systems follow a simple anatomy. The possibility of additional or complex root canal systems should always be considered. This paper reports a mandibular first premolar and a maxillary canine that are single-rooted and have two canals each. Our purpose in reporting this case is to add further information to the clinical evidence for dental professionals about the abnormalities in the morphology of teeth and to highlight the importance of exercising caution when assessing the possibility of additional canals.
